# Eruptive xanthomas and acute pancreatitis in a patient with hypertriglyceridemia

**DOI:** 10.1186/1755-7682-1-6

**Published:** 2008-05-12

**Authors:** Desirée Pérez Martínez, Juan Óscar Fernández Díaz, Carmen Maciá Bobes

**Affiliations:** 1 Internal Medicine Department. Hospital San Agustín. Avilés, Asturias, Spain; 2 Endocrinology Department. Hospital San Agustín. Avilés, Asturias, Spain

## Abstract

Acute pancreatitis and eruptive xanthomas are the only recognised direct complications of severe hypertriglyceridaemia. We present the case of a 33-years old male patient in whom the onset of a type 2 diabetes, added to an unknown familial hyperlipidemia, precipitated a dramatic raise of serum triglyceride levels, that cause in turn an acute pancreatitis and the appearance of dermic eruptive xanthomas.

**Translation:**

This article is translated from Spanish, originally published in *Archivos de Medicina*. The original work is at doi:10.3823/001

## Introduction

Severe hypertriglyceridemia, with higher levels than 2000 mg/dL, can cause the deposit of lipids in the dermis (eruptive xanthomas) and in the retina (*lipemia retinalis*). These symptoms are often described in medical literature, but their observation in clinical practice is rare [1]. Moreover, serum triglyceride levels above 1000 mg/dL are a well-known cause of acute pancreatitis, and in 50% of cases these are associated with mild hyperglycemia [2]. On the contrary, the onset of a diabetes mellitus can produce severe hypertriglyceridemia which develops severely if these lipids show an additional elevation of genetic origin [3]. The clinical case below describes a patient with hyperglycemic symptoms, sudden appearance of abdominal pain and yellowish papules [4].

## Clinical case

**Figure 1:**
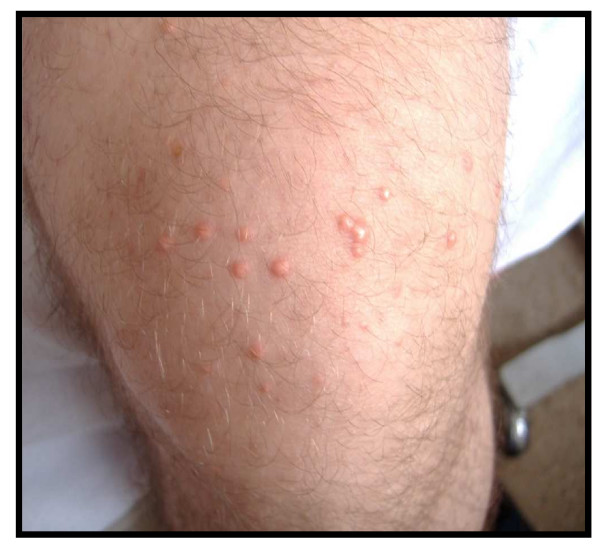
Scattered yellow and white papule bursts on the front of the thigh.

A 33-year-old male, with no history of hereditary dyslipidaemia or diabetes, obese, teetotal, was admitted to hospital with a clinical picture of acute pain in the left hypochondrium and vomiting. Furthermore, in the last 10 days he was presented with papular dermatosis and diabetic symptoms. Clinical exploration with deep pressure elicited pain in the upper part of the abdomen, but there were no peritonitis symptoms. Scattered white-yellowish papules burst on the lower limbs, buttocks and thorax (figure 1).

**Figure 2:**
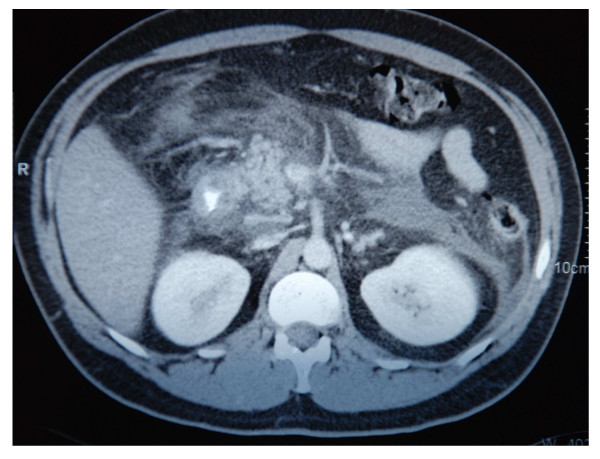
Axial computerized tomography: edematous pancreatitis, without necrosis foci, abscesses, hemorrhage, pseudocysts, associated with liquid deposits in the pararenal region.

Further observation revealed left deviation leukocytosis, a rise of C-reactive protein, glucosuria and ketonuria, hyperglycemia (310 mg/dL), an increase of serum amylase and lipase, extrahepatic cholestasis, and a notable elevation of triglycerides (2350 mg/dL). The axial high-resolution computerized tomography showed an edematous pancreas, in absence of necrosis foci, abscesses or hemorrhage, as well as liquid deposits in the pararenal region (figure 2).

**Figure 3:**
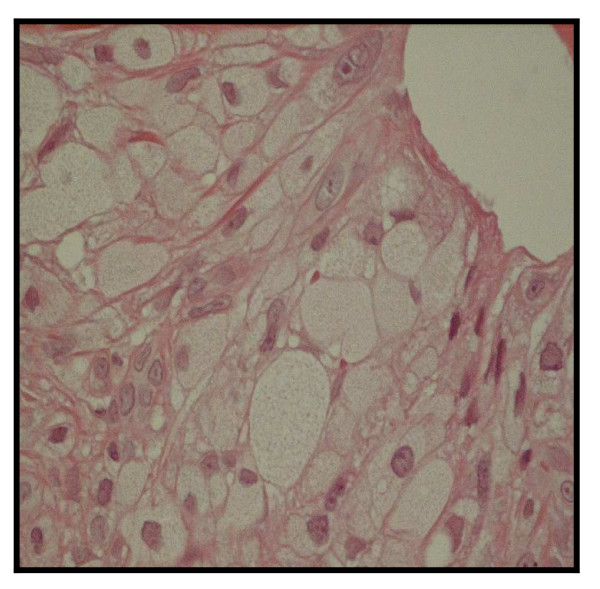
Skin biopsy: clusters of histiocytes with froth-like cytoplasm (hematoxiline-eosine).

The skin biopsy showed dermic bursts of frothy cells, suggestive of a clinical diagnosis of eruptive xanthomatosis (figure 3).

The patient was diagnosed with acute pancreatitis due to hypertriglyceridemia, dermic xanthomatosis and the onset of a type 2 diabetes, with ketosis secondary to vomiting. He received treatment with saline solution, intravenous insulin and analgesia. Pain and hyperglycemic symptoms were controlled rapidly, but subsequent treatment with fibrates was necessary to remove the xanthomas.

## Discussion

Dermic eruptive xanthomatosis is a typical but rare sign of acute hypertriglyceridemia. It manifests itself with sudden appearance of yellowish papules surrounded by a 1–4 mm wide erythematous halo. These are firm and they are normally located on the buttocks and extensor areas of extremities. Histologically, xanthomas are identified by the presence of froth-like histiocytes, containing mainly triglycerides [4,5]. With regard to lipid metabolism, they can be associated with high levels of chylomicrons or with high levels of very low density lipoproteins in serum. Eruptive xanthomas are typical of type-I hyperlipoproteinemia (HLP) (congenital lipoprotein lipase deficit) and type-V HLP (familial combined hyperlipidaemia). They are also known to be associated with type-IV HLP (familial endogenous hypertriglyceridemia) and type-III HLP (serum remnant lipoproteins disease). There are also secondary forms of hyperchylomicronemia, amongst them uncontrolled diabetes mellitus and alcohol consumption [1].

The causes for acute pancreatitis are varied but biliary calculi and alcohol intake feature in 90% of cases. A rare etiology (2–10%) is hypertriglyceridemia [2]. Its pathogenic mechanism is yet unknown, although it has been suggested it could be the toxic effect of free fatty acids and of lysolecithin on cell membranes. These free fatty acids would be generated inside the pancreas due to the effect of pancreatic lipase on triglycerides [6].

The initial treatment of this type of pancreatitis is straightforward, except for perhaps specific parental nutrient solutions (should this nourishing means be necessary). The treatment of the underlying hyperlipidemia tends to require specific drugs, normally fibrates, in order to control it [7]. The case patient received treatment with fenofibrate, started fifteen days after being admitted to hospital. At this moment it was tested that euglycemia was insufficient for the settling of triglycerides (647 mg/dL). As a consequence, the xanthomas had not yet disappeared.

## Authors' contributions

DPM carried out the bibliographic revision and the clinical case description, and participated in the writing of the discussion. JOFD was responsible for the graphic material, and contributed towards the discussion. CMB conceived of the study and participated in its coordination. All authors read and approved the final manuscript.
